# P-1826. Trends in hepatitis C virus laboratory testing in a large, U.S. commercial laboratory, 2018-2024

**DOI:** 10.1093/ofid/ofaf695.1995

**Published:** 2026-01-11

**Authors:** Min Kyung Lee, Charles M Walworth, David Alfego, Laura Gillim

**Affiliations:** Labcorp, Durham, NC; Monogram Biosciences/LabCorp, Laguna Beach, CA; Labcorp, Durham, NC; Labcorp, Durham, NC

## Abstract

**Background:**

The Centers for Disease Control and Prevention (CDC) augmented guidance in 2020 to include recommendations for hepatitis C virus (HCV) screening at least once in a lifetime for all adults. In 2023, recommendations by the CDC emphasized the importance of reflexive HCV antibody (HCV Ab) testing where HCV RNA testing is automatically performed on HCV Ab positive samples. We investigated trends in HCV testing in a large commercial laboratory from 2018 to 2024 to evaluate the impact of recently updated HCV guidelines and laboratory stewardship initiatives.

**Figure 1.**
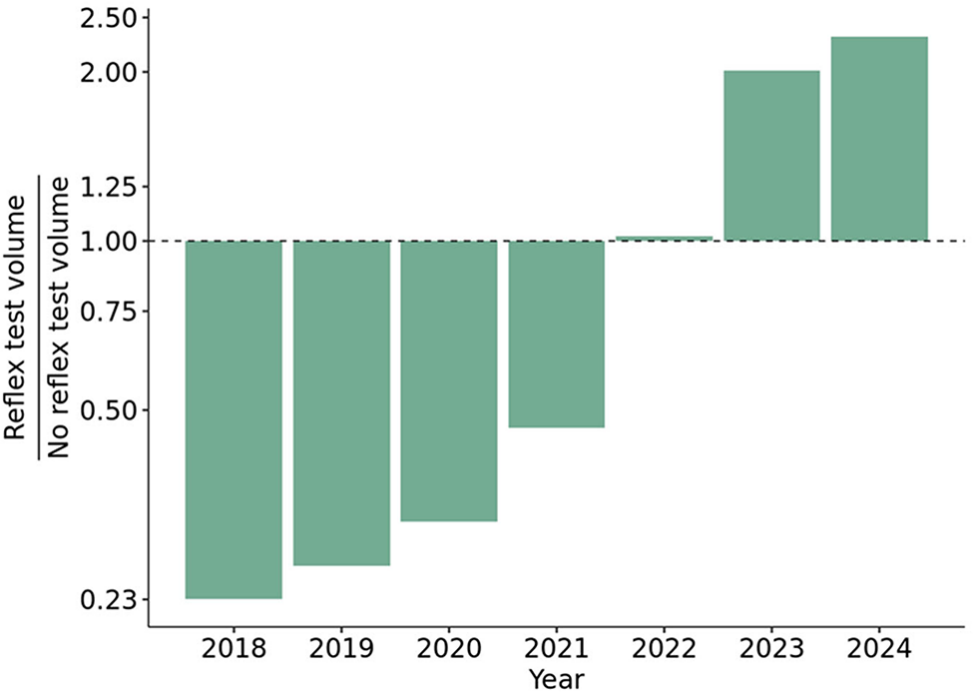
Increased utilization of reflexive HCV antibody testing from 2018 to 2024.

**Figure 2.**
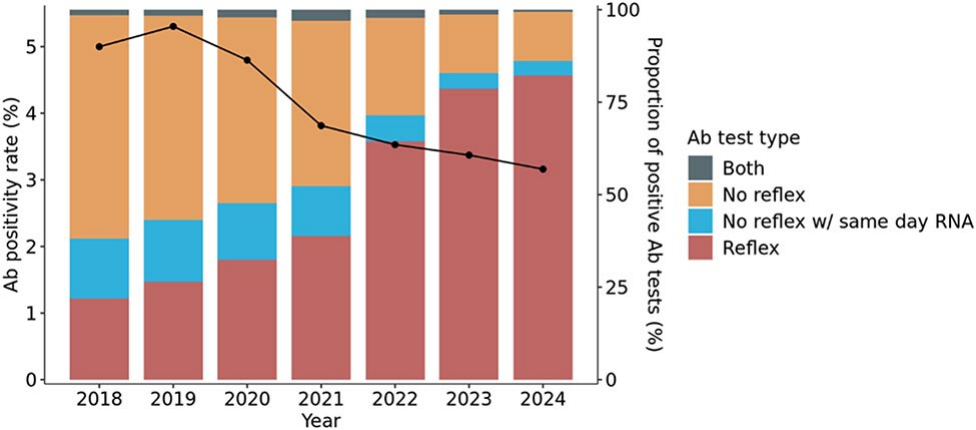
Decreased HCV antibody positivity rates from 2018 to 2024.

**Methods:**

We conducted a retrospective review of 38,233,788 specimens tested for HCV Ab and 1,423,543 follow up HCV RNA tests from January 2018 to December 2024. Test offerings during this period included standalone HCV Ab, standalone HCV quantitative RNA, and a reflexive HCV Ab option. The laboratory communicated the recommended use of reflexive HCV Ab testing to clients in July 2021 and removed the standalone HCV Ab test from the test directory in October 2022.

**Figure 3.**
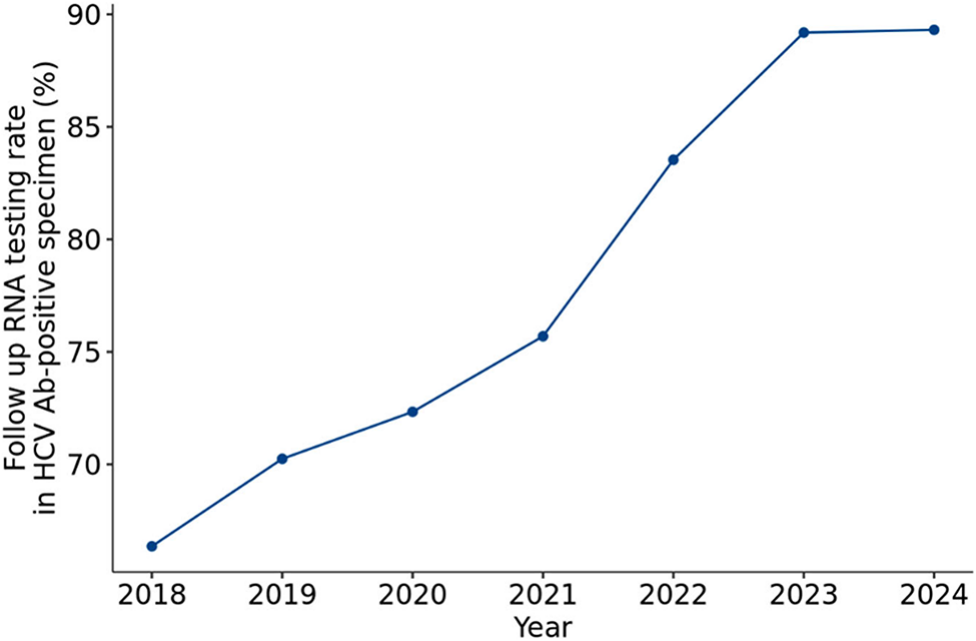
Increased follow up RNA testing rates in HCV Ab-positive specimen from 2018 to 2024.

**Figure 4.**
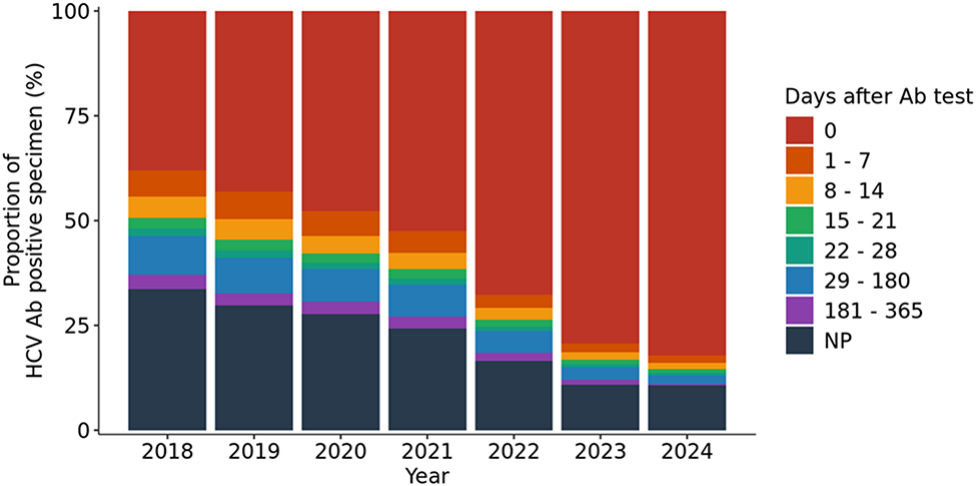
Decreased time to follow up HCV RNA test in HCV Ab-positive specimen from 2018 to 2024.

**Results:**

HCV Ab testing from 2018 to 2020 was constant but steadily increased from 2020 to 2024. The earlier time period had more standalone HCV Ab tests than the latter, although overall reflexive HCV Ab testing increased steadily from 2018-2024 (T=1.00, p=0.003). HCV Ab positivity rates steadily declined from 5.0% in 2018 to 3.2% in 2024 (T=0.0, p=0.007). Among HCV Ab+ individuals, follow-up RNA testing has increased over time (T=1.00, p=0.003), averaging 78.1% during the study period, but increasing to 89% in 2023-2024. Among those HCV Ab+ for whom standalone HCV Ab testing was ordered, follow-up RNA testing averaged 46.8%, whereas HCV RNA testing was completed on the same day in 93.6% of those with reflexive HCV Ab testing. Same day HCV RNA testing on HCV Ab+ samples occurred for 38.0% of tests in 2018 and increased to 82.2% in 2024. Overall HCV RNA positivity decreased 19.2% during this period (figure not shown).

**Conclusion:**

Our study suggests increasing compliance with updated CDC recommendations for HCV screening. Recommendations emphasizing the use of reflexive HCV Ab testing and laboratory stewardship initiatives implemented by the laboratory have increased clinician utilization of reflexive testing and shortened the time to detection of active HCV infection.

**Disclosures:**

Min Kyung Lee, PhD, Labcorp: Employee|Labcorp: Stocks/Bonds (Public Company) Charles M. Walworth, MD, Labcorp: Employee|Labcorp: Stocks/Bonds (Public Company)|Labcorp: Stocks/Bonds (Public Company) David Alfego, PhD, Labcorp: Employee|Labcorp: Employee|Labcorp: Stocks/Bonds (Public Company) Laura Gillim, PhD, Labcorp: Stocks/Bonds (Public Company)

